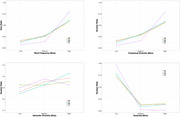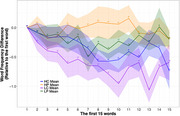# Verbal fluency and distribution analysis for mild cognitive impairment detection

**DOI:** 10.1002/alz70855_105641

**Published:** 2025-12-24

**Authors:** Ya‐Ning Chang, Yi‐Hsuan Wang, Chia‐Ju Chou, Yi‐Chien Liu, Matt Lambon Ralph

**Affiliations:** ^1^ Miin Wu School of Computing, National Cheng Kung University, Tainan, Taiwan; ^2^ College of Medicine, National Cheng Kung University, Tainan, Taiwan; ^3^ Cardinal Tien Hospital, New Taipei City, Taiwan; ^4^ Medical school of Fu‐Jen University, Taipei, Taiwan; ^5^ Tohoku University, Sendai, Japan; ^6^ University of Cambridge, Cambridge, United Kingdom

## Abstract

**Background:**

Verbal fluency is commonly used to differentiate patients with mild cognitive impairment (MCI) from healthy controls (HC). MCI patients generally are less proficient and produce fewer words within a semantic category than HC. However, this approach may be unreliable when HC and MCI exhibit similar word production efficiency. Therefore, in this study, we investigate whether distribution analysis of lexical properties in individual words and within word lists could be useful in detecting MCI with high verbal fluency.

**Method:**

For a verbal fluency task, eighty native Chinese speakers (40 HCs and 40 patients with MCI) were asked to generate words in the Animal category within one minute. For each participant group, we further divided the participants into two subgroups based on word generation proficiency (i.e., number of words), resulting in four groups: high‐proficiency controls (HC), low‐proficiency controls (LC), high‐proficiency patients (HP) and low‐proficiency patients (LP). We then conducted distribution analysis to investigate word properties, including word frequency, contextual diversity, semantic diversity, surprisal, and frequency variation in the word lists.

**Result:**

**T**he distribution analyses (Figure 1) demonstrated significant differences between the control and patient groups (*ps* < 0.05). However, the difference was primarily driven by the LP group, with only a subtle difference between the LC and HP groups. Figure 2 showed frequency variation of the first 15 words in the word list. Critically, there was a clear difference between the control and patient groups. Specifically, the LC group tended to begin the word list with high‐frequency words, with a subsequent decrease in frequency. In contrast, the HP group tended to produce words in a similar frequency band at both the beginning and end of their lists.

**Conclusion:**

The distribution analysis of word properties on individual words could only distinguish the LP group from the others. Differentiation between the LC and HP groups was possible only when frequency variation in the word lists was considered. These observations suggest that MCI patients may exhibit differences in how they structure their semantic memory. However, a larger sample size is needed to further assess the effectiveness and generalisation of this positional word frequency approach.